# Identification of semester-specific teaching contents for dental ethics: development, testing and validation of a questionnaire

**DOI:** 10.1186/s12909-021-02541-x

**Published:** 2021-02-17

**Authors:** Katja Goetz, Ann-Christine Gutermuth, Hans-Jürgen Wenz, Dominik Groß, Katrin Hertrampf

**Affiliations:** 1 Institute of Family Medicine, University Hospital Schleswig-Holstein, Campus Lübeck, Lübeck, Germany; 2 Clinic of Oral and Maxillofacial Surgery, University Hospital Schleswig-Holstein, Campus Kiel, Kiel, Germany; 3 Clinic of Prosthodontics, Propaedeutics and Dental Materials, University Hospital Schleswig-Holstein, Campus Kiel, Kiel, Germany; 4 Institute for History, Theory and Ethics of Medicine, University Hospital Aachen, RWTH Aachen University, Aachen, Germany

**Keywords:** Ethics, Dental education, Validation, Questionnaire

## Abstract

**Background:**

Although medical ethics is an indispensable part of dental education, it has not played a relevant role in the dental curriculum thus far. This study is aimed at developing and validating a questionnaire that identifies semester-specific ethical issues, in order to develop longitudinal ethic modules.

**Methods:**

March 2018 a workshop on item generation was coordinated, using Delphi method; followed by a cognitive testing with students (2nd, 4th, 10th semesters, *n* = 12). A pilot test was carried out with students from different semesters (*n* = 60). The distribution of response frequencies and missing values were determined. The questionnaire used for validation consisted of three dimensions: ethical knowledge, dealing with ethical issues, expectations in terms of teaching. The psychometric examination was carried out by preclinical students (*n* = 105) and clinical semesters (*n* = 110) January 2019.

**Results:**

After cognitive testing and piloting, some items were reformulated, so that a questionnaire with 127 items was used for validation. The individual dimensions were assigned to various factors with excellent to acceptable internal consistency (Cronbach’s α 0.72–0.96).

**Conclusion:**

The questionnaire has an acceptable to excellent consistency and suggests that the different dimensions are conclusive. With this questionnaire, ethical issues in dentistry can be mapped and teaching contents identified.

**Supplementary Information:**

The online version contains supplementary material available at 10.1186/s12909-021-02541-x.

## Introduction

The subject of medical ethics and the discussion of ethical topics and casuistics are now internationally regarded as an integral part of medical education, and they are core elements of the medical curriculum [1–4]. In Germany, medical ethics has gained in importance, at the latest since the turn of the millennium, and it is increasingly regarded as an integrative part of medical education [5, 6]. This led to the decision to make ethics an obligatory part of education in the form of a cross-sectional area in the 9th amendment to the Medical Licensing Regulations 2003 [7].

Studies from the USA, Canada and the United Kingdom showed that the development of specific ethical teaching contents within the sphere of dentistry, and the integration thereof into a curriculum, did not take place at the same time as, and in coordination with that in the sphere of medicine, but followed later [8–11].

In Germany, the educational programs in medicine and dentistry are taught separately except for the natural science subjects. Therefore, there is no transfer of teaching. With regard to dental education, there is still a lack of consideration of ethical learning content in the Dental Licensing Regulations; but that’s also because the 1955 licensing ordinance wasn’t amended until 2019. Accordingly, dental teaching is currently in a transformation phase.

Up to now, in Germany, discussions about integrating the subject or at least an ethics module into the dental curriculum has only been conducted and implemented on a selective basis. So far, only RWTH Aachen University and the Hannover Medical School have included the subject “Dental ethics” in their curricula; here the teaching load is twelve hours, with a focus on basic ethics and case discussions. The rather marginal implementation of the specialty in dental education is due to the partial assumption that dentistry is less affected by ethical questions than medicine is, as the need for vital and dangerous treatment is rare. However, this assessment was commented on by representatives of bioethics [12–15].

The Federal Council’s decision of June 2019 will make it possible to integrate teaching content on medical ethics into dentistry studies in Germany for the first time, by integrating it into a new cross-sectional subject [16]. Due to the pandemic, this modified dental curriculum will not start until winter 2021. The written state examination, which will then be introduced for the first time, will also include questions on ethics.

The integration of ethics into this course of dental study is all the more important, since there are a number of relevant subject areas that require a specific ethical discourse on dentistry [17–19]. This applies to specific aspects of communication between dentist and patient, the mouth or oral cavity – which is also the subject of dental treatment [14, 20]; communication with vulnerable patients and their treatment [21, 22]. Furthermore, there is a high proportion of anxious patients in dentistry, which necessitates the discussion of clinical-practical and situation-related ethical issues and the associated ethics in dentistry.

In a national survey of preparatory assistants, which was conducted two years after the state examination in dentistry, it was shown that ethical aspects are indeed regarded as relevant in the daily work of dentists, and at the same time, they were hardly taken into account [23]. A qualitative needs analysis carried out by members of the group of authors, showed that the participating students in the 6th and 10th semesters had an inadequate theoretical basis in ethics, and therefore, were unable to establish a sufficient relationship to possible questions, for example in patient treatment during the course of treatment [24]. This led to the following question: at which point during studies and in what form should ethics be dealt with?

The controversial discussion described, regarding the form and implementation of ethical teaching contents within medicine, with in terms of the required attitude towards ethical questions among students and future professionals, underlines the necessity of discussions on teaching contents and teaching methods [1, 25–27]. The sole integration of ethical teaching content does not necessarily lead to an increased sensitisation of students of medicine and dentistry [28–30]. Due to this very selective education in ethics in dental education and the heterogeneity of the German federal state system, there is no adequate documentation of what content is taught in ethics by a dental school and to what extent.

Not only at dental schools, but also in the current research literature, a discussion has flared up around the topic of “Ethics in Dentistry”: Marti et al. (2019) recently published a review based on 248 articles. They found “limited evidence of a clear impact, either short-term or long-term, of humanities education in predoctoral dental education. Reflections on humanistic education in the practice of clinical dentistry were sparse” [31]. This review was subsequently reviewed by Alexander Holden (2020), illustrating that the topic is a current research desideratum. Holden sums up, that there is “a clear need for more integration of the humanities within clinical components of dental education, rather than parallel and separate to clinical learning” [32]. Evidence exists to show that the incorporation of the humanities into predoctoral dental education enhances educational outcomes.

Against this background, the aim of the project was to develop and validate a questionnaire with dental students of the School of Dentistry, Kiel, identifying the required course contents and courses in the field of ethics in dentistry, oriented to the respective requirements of each semester.

## Material and methods

The development of the questionnaire was based on the steps recommended for the development of a questionnaire, according to Kallus [33]. A secondary analysis was performed with a qualitative study that had already been conducted with dental students [24], in order to generate themes and ideas for the new questionnaire. This analysis of qualitative data presented a methodology in social research, for the investigation of additional research [34].

As a subsequent step, a workshop was conducted in March 2018, consisted of five people with different qualifications (medical/dental ethicists, test theorist, methodologists (qualitative design/didactics), dental didactician, and dental student). The aim of the workshop was to discuss and define the areas, ‘purpose of the questionnaire’, ‘target group’ and ‘expectations in terms of the results’, and ended with the definition of the response dimensions. Three main dimensions were identified: “previous knowledge in terms of ethical issues”, “dealing with ethical issues”, and “expectations and desires in terms of teaching medical ethics”.

### Development of items

After the workshop, two members of the working group (project leader, test theorist) developed the items and converted them into an appropriate format for a Delphi round.

Each item was listed individually and supplemented with a comment field (consent, no consent, reason for non-consent, alternative proposal). The answer option (consent) and the scaling (six-level) were identical for all items, and they were also matched. The dimension ‘previous knowledge of ethics’ consisted of sixteen items.

The second dimension ‘Dealing with ethical issues’ contained ninety items. The subsequent third dimension ‘Expectations and desires in terms of teaching in medical ethics’ consisted of twenty items. Finally, the participants of the working group were asked to comment on the questionnaire used in the qualitative study on sociodemographic and, for example, on the question of volunteer work.

A total of thirty-nine items were commented on. Concrete suggestions for reformulation were made for twenty-three items. The remaining sixteen items were editorial comments. These comments were entered for a second Delphi round, and then circulated within the working group once again. The members of the working group unanimously approved the proposed changes.

Since students were involved in the next steps, the study protocol for the testing and validation of the ethics commission was presented. The project was approved by the Ethics Committee of the University of Kiel, Germany (D477/18).

### Cognitive test

The review of the items was carried out by means of cognitive interviews, via the ‘concurrent think aloud protocol’ [35] with the aim of identifying problems and difficulties in terms of the intelligibility and comprehensibility of the items, documentation with problems in reply to the items and their ranking within the questionnaire, documentation of interests and awareness of the items, and the documentation of technical problems encountered when completing the questionnaire.

This cognitive test was carried out with dental students, four each from the 2nd, 4th and 10th semesters and took place in the summer semester of 2018. Participation in the study was voluntary. The inclusion criteria were the admission to one of the above-mentioned semesters, a written letter of informed consent and sufficient knowledge of the German language to take part in the interview. After agreeing to participate, a personal interview time slot was arranged with each student. In case of more volunteer students, a randomised selection was carried out per draw. All interviews were carried out by the same member of the working group.

Prior to the interview, a short questionnaire was filled in, with regard to the sociodemographic background, voluntary work or potential prior experience with a personal event that raised ethical questions. Each interview was recorded under standardised headings, such as ‘problems encountered’, ‘disruption during the interviews’ and ‘time required’. The questionnaire and the interview were coded identically for all students. No code list was conducted; therefore, it was not possible to conclude which student was involved in which interview (anonymised).

Each interview was recorded with a digital audio device and the interview was transcribed according to the formulated transcription rules. A qualitative content analysis was conducted, and problems in terms of comprehension and replies to the items were systematically evaluated.

### Pilot test of the questionnaire

The pilot test was conducted with preclinical dental students at the same School of Dentistry, from the 1st, 3rd and 5th semesters (*n* = 25–30), and with clinical dental students who did not take part in the cognitive test, from the 6th to the 10th semesters (*n* = 25–30).

At the School of Dentistry, Kiel, the accreditation of the course of studies is only possible in the winter semester. As a consequence, in the winter semester, there are only 1st, 3rd and 5th semester courses, with around sixty-six students in each semester. After the first state exam (after the 5th semester), the accreditation switches from once a year to twice a year (summer and winter semester). Therefore, the number of students in one preclinical semester are divided in two clinical semesters.

The recruitment strategy and the inclusion criteria were the same for the cognitive test, which was carried out at the beginning of the winter semester 2018/2019. The willingness of the 1st and 5th semester students to participate was greater than the desired ten participants per semester. Here, ten students were drawn at random. After agreeing to participate, different time slots were offered for the completion of the questionnaire.

#### Statistical analysis

The analysis was performed using the statistical programme SPSS 25.0 (SPSS Inc., IBM).

The evaluation strategy included checking the feasibility of the items (behaviour of the respondents when filling in the form) concerning the recommendation by Kelley [36]. A descriptive analysis on the distribution of response frequencies and the determination of the rate of missing values was performed.

### Psychometric properties of the final questionnaire

The recruitment of the students took place in January 2019. The students of each semester were informed by two members of the working group (Preclinic HJW, Clinic KH) and invited to participate. The project group aimed at having a sample of dental students, divided into the pre-clinical semesters, 1st, 3rd and 5th semesters (35 students each) and the clinical semesters, 6th to 10th semesters (ideally, the full survey). The recruitment strategy was in accordance with recommendations by Terwee [37]. The participating students filled in the questionnaire and, in addition, they also answered the sociodemographic questions that had already been used. Please see the final questionnaire in additional file 2.

#### Statistical analysis

The analysis was performed using the statistical programme SPSS 25.0 (SPSS Inc., IBM).

The evaluation strategy included psychometric testing by means of a descriptive analysis, including an analysis of response distribution and item difficulty. For item difficulty, values from 20 to 80% were preferred. Values from 0 to 20% were interpreted as extremely difficult and values from 80 to 100% were interpreted as extremely easy [38].

**Fig. 1:**
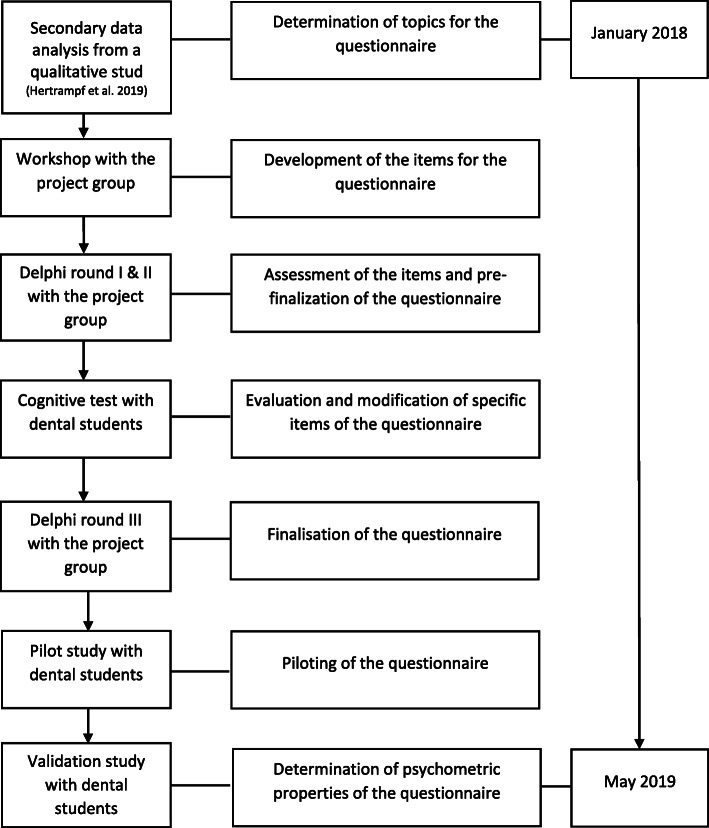
Flowchart of the development steps of the questionnaire

Furthermore, principal component analysis with extraction of component loadings was performed. The component loadings were subjected to Varimax rotation, and their number was determined by eigenvalues > 1. Furthermore, sample suitability was evaluated according to the Kaiser-Meyer-Olkin (KMO) criterion, and Bartlett’s test was performed to examine sphericity (*p* < 0.05) [39]. All items with a component loading > 0.4 were assigned to the particular component. Internal consistency was determined by Cronbach’s α [40]. Values > 0.9 are regarded as excellent and > 0.8 represent good internal consistency, while values > 0.7 and values > 0.5 represent acceptable consistency and poor internal consistency respectively [41]. An overview of the development process, under consideration of the various steps, is presented in Fig. 1.

## Results

### Item development

The participants of the workshop defined the areas ‘purpose of the questionnaire’, ‘target group’, and ‘expectations of the results’. The purpose was to ‘define, develop and impart teaching content in a way that is appropriate for longitudinal students’. The term ‘module’ was deliberately omitted, as it already represents a definition of the form of implementation. The target group was defined as ‘dental students of all semesters’. The working group defined the ‘identification of ethical reference points in studies’ and the ‘identification of psychometric characteristics (questionnaire)’ as expectations in terms of the results. With regard to the possible response alternatives (frequency, intensity, probability, agreement), the working group decided that no specific response alternatives would be excluded due to the dimensions and items that have not yet been defined. However, for an uncomplicated answer, it should be considered that not too many different alternatives should be included in the questions. With regard to answer scaling, consensus was reached on avoiding a ‘do not know’/medium category. Based on the results of the qualitative study, an initial possible classification of the questionnaire was determined as follows:

- Content of the 1st dimension, which was called “Previous knowledge regarding ethical issues”
- Content of the 2nd dimension, which was called “Dealing with ethical issues”
- Content of the 3rd dimension, which was called “Expectations and desires in terms of teaching medical ethics”

### Cognitive test

Twelve dental students were willing to participate. The test conducted showed no problems with regard to answering the questionnaire in general, in relation to the formulation of the items, their order within the questionnaire or technical problems when filling out the questionnaire.

In concrete terms, the majority of the students expressed comprehensibility problems with individual terms and, as a result, they suggested explanations or definitions. This concerned terms such as ‘vulnerable’, ‘intercultural conflict’, ‘stigmatisation’, ‘overuse’, ‘patient representative’, ‘POL’ and ‘clinical ethics advice’. Definitions of these terms were added within the body of the questionnaire, partly with examples, and agreed upon within the working group, within the framework of a Delphi round.

### Pilot test

A total of sixty students took part. For one participant, the sociodemographic data was missing. Further details are presented in Table 1. The analyses of the feasibility of the items showed a wide distribution of response frequencies and hardly any missing values. No ceiling and floor effects were determined. Therefore, no items were deleted from the questionnaire.

**Table 1:** Characteristics of the study population – pilot (*n* = 60) and validation study (*n* = 215)

Variables		Pilot study; Number (%)	Validation study; Number (%)
**Gender n (%)**	**Female**	42 (70.0%)	150 (69.8)
	**Male**	17 (28.3%)	65 (30.2)
**Age, mean (SD); range**		23.5 (3.0); 18–33	25.3 (4.1); 19–44
**Voluntary work, yes**		14 (23.3)	38 (17.7)
**Jobbing in addition to study, yes**		25 (41.7)	89 (41.4)
**Organ donor card filled in, yes**		34 (56.7)	112 (52.1)
**Semester of study, mean (SD); range**		5.6 (3.03); 1–11	5.6 (3.0); 1–10

### Psychometric properties of the final questionnaire

A total of 215 dental students participated in the final survey, of whom 150 (69.8%) were female and sixty-five (30.2%) were male. The average age was 25.3 years (SD 4.1). Further sociodemographic information is presented in Table 1.

#### Dimension: previous knowledge regarding ethical issues

The distribution of the data for the sixteen items was not normal. The analyses of the item difficulties showed values from 31.3% (‘Ethicists are unacquainted with medical and clinical issues’) to 70.7% (‘Morality is understood to mean social norms and values that guide the actions of society’). The principal component analysis revealed a four-component solution with a total variance (R^2^) of 55.5% (KMO = 0.71, Bartlett’s test for sphericity *P* < 0.001). The Cronbach’s α value for internal consistency was 0.721 (please see in detail supplementary Table 1).

#### Dimension: dealing with ethical issues

This dimension was divided into five scales.

The distribution of the data of the ‘knowledge’ scale for the eighteen items was not normal (please see in detail supplementary Table 2).

The mean values of the individual answer options ranged from 0.99 (‘During my studies, I was taught how to deal with intercultural conflicts’) to 3.27 (‘During my studies, I was taught what dental confidentiality entails’). The item difficulties were acceptable, with the expectation of the item ‘During my studies, I was taught how to deal with intercultural conflicts’ of 19.8%. The principal component analysis revealed a three-component solution with a total variance (R^2^) of 66.8% (KMO = 0.94, Bartlett’s test for sphericity *P* < 0.001). The Cronbach’s α value for internal consistency was 0.944.

The ‘estimation of importance’ scale is presented in supplementary Table 3. The distribution of the data for the eighteen items was not normal. Nearly every item showed an item difficulty that was interpreted as extremely easy to answer. The principal component analysis revealed a three-component solution with a total variance (R^2^) of 68.8% (KMO = 0.93, Bartlett’s test for sphericity *P* < 0.001). The Cronbach’s α value for internal consistency was 0.868.

The distribution of the data ‘personal experiences’ scale for the eighteen items was not normal (please see in detail supplementary Table 4).

With the exception of three items, the item difficulties were acceptable. The items ‘I have my own experience with parents who refuse to have their child treated’, ‘I have my own experience in terms of stigmatisation in medicine’, and ‘I have my own experience with the involvement of patient representatives’ showed item difficulties that were interpreted as ‘extremely difficult to answer’. The principal component analysis revealed a four-component solution, with a total variance (R^2^) of 66.7% (KMO = 0.91, Bartlett’s test for sphericity *P* < 0.001). The Cronbach’s α value for internal consistency was 0.923.

The distribution of the data ‘safety / uncertainty’ scale for the seventeen items was not normal (please see in detail supplementary Table 5).

The item difficulties were acceptable for the expectations in terms of the item ‘I feel confident in dealing with parents who refuse to have their child treated’ (17.9%). The principal component analysis revealed a three-component solution with a total variance (R^2^) of 71% (KMO = 0.93, Bartlett’s test for sphericity *P* < 0.001). The Cronbach’s α value for internal consistency was 0.952.

The distribution of the data ‘need for support’ scale for the seventeen items was not normal (please see in detail supplementary Table 6). Three items showed a level of difficulty that was interpreted as extremely easy. These were ‘I would like support in dealing with treatment errors’, ‘I would like support in dealing with patients who can no longer make their own decisions’, and ‘I would like support in dealing with parents who refuse to have their child treated’. The principal component analysis revealed a two-component solution with a total variance (R^2^) of 69.8% (KMO = 0.94, Bartlett’s test for sphericity *P* < 0.001). The Cronbach’s α value for internal consistency was 0.96.

#### Dimension: expectations and desires in terms of teaching medical ethics

The distribution of the data for the twenty-two items was not normal (please see in detail supplementary Table 7). Four items showed values that were interpreted as extremely easy to answer. The principal component analysis revealed a five-component solution with a total variance (R^2^) of 63.7% (KMO = 0.89, Bartlett’s test for sphericity *P* < 0.001). The Cronbach’s α value for internal consistency was 0.914.

An overview of the different dimensions and scales is given in Table 2.

**Table 2:** Overview of the dimensions of the final questionnaire

Dimensions	Scales	Number of items	Cronbach’s alpha
Previous knowledge regarding ethical issues		16	0.721
Dealing with ethical issues	Knowledge	18	0.944
	Estimation of importance	18	0.868
	Personal experience	18	0.923
	Safety / uncertainty	17	0.952
	Need of support	17	0.963
Expectations and desires regarding the teaching of medical ethics		22	0.914

## Discussion

The newly developed questionnaire is comprehensive, manageable and easy to analyse. It enables a differentiated consideration of ethical issues during dental education. The development process of the questionnaire are in accordance with suggestions by Kelley [36]. The participants of pilot group are comparable to the participants of the validation study concerning the different sociodemographic variables. Despite the extensive number of items, hardly any missing data was observable.

The internal structure of the dimensions with respect to the scales of the 2nd dimension, was tested by means of reliability and factor analysis testing. The dimension ‘previous knowledge regarding ethical issues’ and ‘expectations and desires in terms of teaching medical ethics’ achieved acceptable internal consistencies, as did the different scales from the dimension ‘dealing with ethical issues’.

The scale ‘estimation of importance’ from the dimension ‘dealing with ethical issues’ shows various items with an item difficulty that was interpreted as extremely easy to answer. It should be considered whether this scale is necessary for the questionnaire, or whether a reformulation of relevant items would be useful. Considering the scope of the questionnaire, an omission of the scale ‘estimation of importance’ could be a possible solution. It can be assumed that the participating dental students found these different aspects important, therefore no dispersion of the values is observable.

Overall, the questionnaire covered a broad range of ethical issues. These range from communication with vulnerable patients, through conflict situations between dentist and patient, to problematizing a wish-fulfilling (non-therapeutic) dentistry [42]. These topics should be a part of the curriculum, in order to teach students how to deal with these issues in the oral health care of patients. The results of the questionnaire, for example the identification of the level of existing knowledge, provide important information on where and to what intensity these topics can be incorporated into the dental curriculum. This approach beyond established ethical teaching content to implement specific ethical teaching content for dentistry has been detailed in studies and reviews for the development and implementation of an ethical curriculum within the context of dental studies [18, 31, 43, 44]. Thus, the problem that a sole integration of ethical teaching contents into the studies without a corresponding didactic basis does not automatically lead to a sensitization is adressed [28–30]. The questionnaire thereof enables an assessment of expectations and wishes regarding the design of medical ethics teaching. An interesting and important aspect of this is that the students’ assessment and perspective are taken into account [45–48].

Our study also offers evidence that students have a need for ethics training. It is widely recognized that ethical dilemmas regularly arise in medical treatment, and there is no reason to believe that this should be any different in dental treatment. Likewise, we now know that ethics classes in medical school have proven their worth and are considered essential [49]. Accordingly, the same effect can be expected for dentistry. This assumption is also consistent with the conclusion of Holden (2020): He elaborates that Ethics and Humanities in general empower dental students to increase their focus “on hearing the voices of patients in articulating their experience of disease and ill-health”. Holden (2020) rightly emphasizes that “this collaboration of approaches has not yet to be fully realized in the context of dentistry, but initiatives to promote a collaborative approach would have a powerful effect on dental students’ development as humanistic and empathetic practitioners” [32].

In addition, the questionnaire could support the basis for a discourse within medical faculties where structural framework conditions do not allow an automatic transfer of teaching content between the courses of study in medicine and dentistry. Besides, the questionnaire is helpful for clarifying the extent to which established teaching content in medicine could potentially be useful for dental education [50].

Since ethics is to be part of the dental curriculum (with the introduction of the new dental licensing regulations in the winter of 2021) and is also to be a topic in the written state examination for the first time, the questionnaire can provide very concrete assistance with two specific, currently pending decisions.

### Limitations and strengths

The high response rate in the validation study was mainly attributed to the personal conduct to the students by members of the working group. However, the development of the questionnaire took place at a single location, which could have introduced a potential selection bias. The new dental licensing regulations make it necessary to compare the implementation of ethics at the different universities on a national level, which could be the subject of further studies. Furthermore, only internal consistency and factor structure were determined, which reflected no complete reliability analysis concerning the “Consensus-based Standards for the selection of health Measurement Instruments” checklist [51]. Finally, the results of the study were only explorative, and they should be confirmed in further studies, which include a test–retest design.

## Conclusion

However, it can be concluded that the newly developed questionnaire presents a tool for the design and implementation of a longitudinal curriculum that includes the ethical aspect in dental studies and reflects different ethical issues in dental health care. The questionnaire supports and promotes the implementation of the new dental licensing regulations in the field of ethics. Moreover, it helps identifying the most requested teaching contents in ethics and thus contributes to a continuing professional development in dental teaching.

## Supplementary Information

**Additional file 1.**

**Additional file 2.**
