# Supplementary material for: Identification of semester-specific teaching contents for dental ethics: development, testing and validation of a questionnaire
Source: BMC Med Educ. 2021 Feb 17;21:109. doi: 10.1186/s12909-021-02541-x (PMC7890951; doi:10.1186/s12909-021-02541-x)
Supplement: Supplementary file 1 — Additional file 1. [file 12909_2021_2541_MOESM1_ESM.docx]

**Identification of semester-specific teaching contents for dental ethics:**

**Development, testing and validation of a questionnaire**

**-**

**Supplementary information file**

Katja Goetz^1^, Ann-Christine Gutermuth^2^, Hans-Jürgen Wenz^3^, Dominik Groß^4^, Katrin Hertrampf^2^

^1^Institute of Family Medicine

University Hospital Schleswig-Holstein, Campus Lübeck, Germany

^2^Clinic of Oral and Maxillofacial Surgery

University Hospital Schleswig-Holstein, Campus Kiel, Germany

^3^Clinic of Prosthodontics, Propaedeutics and Dental Materials

University Hospital Schleswig-Holstein, Campus Kiel, Germany

^4^Institute for History, Theory and Ethics of Medicine

University Hospital Aachen, RWTH Aachen University, Germany

**Supplementary table 1: Dimension: Previous knowledge regarding ethical issues**

|  | **Mean (SD)** | **Item difficulty** | **KMO** | **Component loading** | | | |
| --- | --- | --- | --- | --- | --- | --- | --- |
|  |  |  |  | **Component 1** | **Component 2** | **Component 3** | **Component 4** |
| So far, there have been no ethical conflicts within the family. | 2.88 (1.76) | 57.6 | 0.71 | 0.73 |  |  |  |
| So far, there has been no situation during my studies in which ethics has played a role. | 2.16 (1.78) | 43.2 | 0.67 | 0.71 |  |  |  |
| So far, there have been no ethical conflicts within my circle of friends. | 2.43 (1.66) | 48.6 | 0.75 | 0.86 |  |  |  |
| Ethicists are unacquainted with regard to medical and clinical issues. | 1.57 (1.27) | 31.3 | 0.70 |  |  | 0.80 |  |
| Ethicists cannot adequately evaluate treatment situations on patients, due to a lack of personal treatment experience. | 2.30 (1.22) | 46.0 | 0.59 |  |  | 0.77 |  |
| Ethicists set moral principles | 2.59 (1.27) | 51.7 | 0.78 |  | 0.60 |  |  |
| Morality is understood to mean social norms and values that guide the actions of an individual. | 3.54 (1.23) | 70.7 | 0.74 |  | 0.42 |  | 0.48 |
| Ethics are social norms and values that guide the actions of society. | 3.14 (1.35) | 62.8 | 0.65 |  | 0.69 |  |  |
| Ethics is a synonym for morality. | 1.63 (1.34) | 32.6 | 0.69 |  | 0.70 |  |  |
| Professional ethics refers to the idea of values in terms of how society views a professional group. | 2.55 (1.39) | 50.9 | 0.70 |  | 0.64 |  |  |
| Professional ethics describes individual moral aspects of a professional group. | 2.79 (1.28) | 55.7 | 0.66 |  |  |  | 0.76 |
| Etiquette describes a set of rules of conduct that describes the expectations placed on the social behaviour of members of a professional group. | 3.30 (1.27) | 66.0 | 0.69 |  |  |  | 0.80 |
| Etiquette is not about ethics. | 3.06 (1.61) | 61.1 | 0.71 |  |  | 0.45 |  |
| **If you now reflect on this complex of questions in retrospect, to what extent do the following statements apply, from your point of view?** |  |  |  |  |  |  |  |
| Ethical conflicts have not played a role in my family so far. | 2.53 (1.75) | 50.7 | 0.73 | 0.79 |  |  |  |
| There have been no situations during my studies in which ethics has played a role. | 2.12 (1.70) | 42.4 | 0.72 | 0.76 |  |  |  |
| Within my circle of friends there have been no ethical conflicts so far. | 2.37 (1.63) | 47.3 | 0.73 | 0.82 |  |  |  |

SD standard deviation; KMO Kaiser-Meyer Olkin; ^#^ range from 0 – disagree entirely to 5 – agree completely; * Loading > 0.4 were reported

**Supplementary table 2: Dimension: Dealing with ethical issues – scale: Knowledge**

| **During my studies I was taught…** | **Mean (SD)** | **Item difficulty** | **KMO** | **Component loading*** | | |
| --- | --- | --- | --- | --- | --- | --- |
|  |  |  |  | **Component 1** | **Component 2** | **Component 3** |
| how to educate the patients. | 2.74 (1.77) | 54.9 | 0.92 |  | 0.81 |  |
| what dental confidentiality entails. | 3.27 (1.73) | 65.4 | 0.92 |  | 0.82 |  |
| how to deal with vulnerable patient groups during treatment. | 1.78 (1.55) | 35.6 | 0.94 | 0.61 | 0.55 |  |
| how to deal with phobic patients. | 1.82 (1.58) | 36.4 | 0.93 | 0.56 | 0.64 |  |
| what a treatment error entails. | 2.51 (1.65) | 50.1 | 0.93 |  | 0.67 |  |
| how to deal with a conflict between dental student and lecturer regarding the therapy decision. | 1.47 (1.53) | 29.3 | 0.97 | 0.65 | 0.42 |  |
| how to deal with patients who can no longer make their own decisions. | 1.33 (1.46) | 26.7 | 0.92 | 0.69 | 0.44 |  |
| how to deal with patients who have a lack of financial possibilities for the recommended therapy. | 1.51 (1.43) | 30.1 | 0.96 | 0.59 |  |  |
| how to deal with claims from patients after therapy without medical indication. | 1.70 (1.53) | 33.9 | 0.95 | 0.52 |  | 0.44 |
| how to deal with parents who refuse to have their child treated. | 1.09 (1.36) | 21.9 | 0.92 | 0.80 |  |  |
| how to deal with intercultural conflicts. | 0.99 (1.23) | 19.8 | 0.92 | 0.87 |  |  |
| how to deal with stigmatisation. | 1.19 (1.42) | 23.7 | 0.94 | 0.79 |  |  |
| to inform about diagnoses truthfully. | 2.33 (1.81) | 46.5 | 0.95 |  | 0.54 | 0.41 |
| how to deal with wish-fulfilling treatment (i.e. aesthetic/cosmetic dentistry). | 2.21 (1.61) | 44.3 | 0.95 |  | 0.48 | 0.51 |
| that a dentist could be the head of a clinical trial. | 2.68 (1.73) | 53.6 | 0.90 |  |  | 0.82 |
| how to deal with uncooperative behaviour. | 1.57 (1.45) | 31.4 | 0.93 | 0.55 |  | 0.57 |
| how to deal with oversupply. | 1.42 (1.39) | 28.4 | 0.93 | 0.49 |  | 0.61 |
| how to deal with the involvement of patient representatives. | 1.12 (1.35) | 22.4 | 0.93 | 0.60 |  | 0.47 |

SD standard deviation; KMO Kaiser-Meyer Olkin; ^#^ range from 0 – disagree entirely to 5 – agree completely; * Loading > 0.4 were reported

**Supplementary table 3: Dimension: Dealing with ethical issues – scale: Estimation of importance**

| **I think it is important that, during my studies, I am taught …** | **Mean (SD)** | **Item difficulty** | **KMO** | **Component loading** | | |
| --- | --- | --- | --- | --- | --- | --- |
|  |  |  |  | **Component 1** | **Component 2** | **Component 3** |
| about the education of patients. | 4.65 (0.85) | 93.1 | 0.90 |  | 0.84 |  |
| about the observation of dental confidentiality. | 4.64 (0.87) | 92.9 | 0.91 |  | 0.81 |  |
| about the truthful information of diagnoses. | 4.64 (0.80) | 92.8 | 0.93 | 0.42 | 0.81 |  |
| about the treatment of vulnerable groups of patients. | 4.50 (0.99) | 89.9 | 0.93 | 0.56 | 0.70 |  |
| about dealing with phobic patients. | 4.56 (0.88) | 91.1 | 0.92 | 0.51 | 0.73 |  |
| about dealing with treatment errors. | 4.75 (0.72) | 95.0 | 0.92 |  | 0.83 |  |
| about dealing with patients who have a lack of financial possibilities for the recommended therapy. | 4.39 (1.02) | 87.8 | 0.92 | 0.59 | 0.56 |  |
| about dealing with patients who can no longer make their own decisions. | 4.34 (1.10) | 86.8 | 0.93 | 0.76 |  |  |
| about dealing with conflict between dental students and lecturer regarding the therapy decision. | 4.11 (1.22) | 82.3 | 0.93 | 0.76 |  |  |
| about dealing with intercultural conflicts. | 3.82 (1.42) | 76.3 | 0.94 | 0.89 |  |  |
| about dealing with claims from patients after therapy without medical indication. | 4.47 (3.66) | 89.4 | 0.87 |  |  | 0.81 |
| about dealing with parents who refuse to have their child treated. | 4.39 (1.06) | 87.8 | 0.94 | 0.74 |  |  |
| that a dentist can be the head of a clinical trial. | 3.23 (1.53) | 64.5 | 0.91 | 0.57 |  |  |
| about dealing with wish-fulfilling treatment (i.e. aesthetic/cosmetic dentistry). | 4.43 (2.92) | 88.6 | 0.87 |  |  |  |
| about dealing with stigmatisation. | 3.82 (1.39) | 76.5 | 0.92 | 0.87 |  |  |
| to deal with uncooperative behaviour. | 3.74 (1.44) | 74.8 | 0.94 | 0.80 |  |  |
| about dealing with oversupply. | 4.06 (1.16) | 81.1 | 0.95 | 0.83 |  |  |
| about dealing with the involvement of patient representatives. | 4.17 (1.11) | 83.3 | 0.96 | 0.79 |  |  |

SD standard deviation; KMO Kaiser-Meyer Olkin; ^#^ range from 0 – disagree entirely to 5 – agree completely; * Loading > 0.4 were reported

**Supplementary table 4: Dimension: Dealing with ethical issues – scale: Personal experiences**

| **I have my own experience …** | **Mean (SD)** | **Item difficulty** | **KMO** | **Component loading** | | | |
| --- | --- | --- | --- | --- | --- | --- | --- |
|  |  |  |  | **Component 1** | **Component 2** | **Component 3** | **Component 4** |
| in educating patients. | 2.73 (1.94) | 54.5 | 0.89 | 0.89 |  |  |  |
| in observing dental confidentiality. | 3.26 (1.90) | 65.1 | 0.90 | 0.83 |  |  |  |
| with phobic patients. | 2.37 (1.91) | 47.3 | 0.91 | 0.84 |  |  |  |
| with vulnerable groups of patients. | 1.88 (1.81) | 37.6 | 0.90 | 0.67 | 0.47 |  |  |
| with treatment errors. | 1.32 (1.51) | 26.4 | 0.95 | 0.44 | 0.47 |  |  |
| with patients who have a lack of financial possibilities for the recommended therapy. | 1.71 (1.80) | 34.1 | 0.93 | 0.67 |  |  |  |
| in terms of conflict between dental students and lecturer regarding the therapy decision. | 1.24 (1.60) | 24.8 | 0.94 |  |  | 0.45 |  |
| with patients who can no longer make their own decisions. | 1.14(1.66) | 22.9 | 0.90 |  | 0.74 |  |  |
| with claims from patients after therapy without medical indication. | 1.06 (1.57) | 21.2 | 0.90 | 0.40 | 0.67 |  |  |
| with parents who refuse to have their child treated. | 0.72 (1.37) | 14.4 | 0.89 |  | 0.82 |  |  |
| with intercultural conflicts. | 1.04 (1.54) | 20.8 | 0.85 |  | 0.79 |  |  |
| with a dentist who is the head of a clinical trial. | 1.21 (1.72) | 24.2 | 0.92 |  |  |  | 0.77 |
| with stigmatisation. | 0.97 (1.48) | 19.3 | 0.89 |  | 0.73 |  |  |
| in dealing with truthfulness (e.g. information about diagnoses). | 2.03 (1.96) | 40.6 | 0.95 | 0.64 |  |  |  |
| with wish-fulfilling treatment (i.e. aesthetic/cosmetic dentistry). | 1.83 (1.83) | 36.7 | 0.95 | 0.70 |  |  |  |
| with uncooperative behaviour. | 2.79 (1.78) | 55.7 | 0.91 |  |  | 0.78 |  |
| with oversupply. | 1.32 (1.54) | 26.3 | 0.92 |  | 0.46 | 0.57 |  |
| with the involvement of patient representatives. | 0.95 (1.50) | 19.1 | 0.92 |  | 0.71 |  |  |

SD standard deviation; KMO Kaiser-Meyer Olkin; ^#^ range from 0 – disagree entirely to 5 – agree completely; * Loading > 0.4 were reported

**Supplementary table 5: Dimension: Dealing with ethical issues – scale: Safety / Uncertainty**

| **I feel confident in…** | **Mean (SD)** | **Item difficulty** | **KMO** | **Component loading** | | |
| --- | --- | --- | --- | --- | --- | --- |
|  |  |  |  | **Component 1** | **Component 2** | **Component 3** |
| the education of patients. | 2.13 (1.79) | 42.5 | 0.94 | 0.78 |  |  |
| the observation of dental confidentiality. | 3.22 (1.73) | 64.4 | 0.89 | 0.50 | 0.73 |  |
| dealing with truthfulness (e.g. information about diagnoses). | 2.91 (1.70) | 58.1 | 0.91 | 0.62 | 0.64 |  |
| the treatment of vulnerable groups of patients. | 1.75 (1.60) | 35.0 | 0.95 | 0.84 |  |  |
| dealing with phobic patients. | 1.79 (1.66) | 35.8 | 0.95 | 0.82 |  |  |
| dealing with treatment errors. | 1.33 (1.42) | 26.6 | 0.93 | 0.81 |  |  |
| dealing with patients who have a lack of financial possibilities for the recommended therapy. | 1.41 (1.45) | 28.1 | 0.96 | 0.85 |  |  |
| dealing with conflicts between dental students and lecturer regarding the therapy decision. | 1.34 (1.37) | 26.9 | 0.96 | 0.79 |  |  |
| dealing with patients who can no longer make their own decisions. | 1.07 (1.26) | 21.3 | 0.91 | 0.80 |  |  |
| dealing with claims from patients after therapy without medical indication. | 1.38 (1.45) | 27.6 | 0.95 | 0.83 |  |  |
| dealing with parents who refuse to have their child treated. | 0.90 (1.21) | 17.9 | 0.93 | 0.77 |  |  |
| dealing with intercultural conflicts. | 1.57 (1.51) | 31.4 | 0.90 | 0.69 |  | 0.60 |
| aesthetic/cosmetic dentistry. | 1.77 (1.61) | 35.5 | 0.89 | 0.73 |  |  |
| dealing with stigmatisation. | 1.67 (1.56) | 33.3 | 0.90 | 0.72 |  | 0.46 |
| dealing with uncooperative behaviour. | 2.09 (1.58) | 41.8 | 0.96 | 0.71 |  |  |
| dealing with oversupply. | 1.16 (1.25) | 23.2 | 0.93 | 0.77 |  |  |
| dealing with the involvement of patient representatives. | 1.14 (1.48) | 22.7 | 0.96 | 0.72 |  |  |

SD standard deviation; KMO Kaiser-Meyer Olkin; ^#^ range from 0 – disagree entirely to 5 – agree completely; * Loading > 0.4 were reported

**Supplementary table 6: Dimension: Dealing with ethical issues – scale: Need for support**

| **I would like support in terms of…** | **Mean (SD)** | **Item difficulty** | **KMO** | **Component loading** | |
| --- | --- | --- | --- | --- | --- |
|  |  |  |  | **Component 1** | **Component 2** |
| the education of patients. | 3.53 (1.53) | 70.5 | 0.95 | 0.78 | 0.43 |
| the implementation of dental confidentiality. | 2.92 (1.72) | 58.5 | 0.94 | 0.68 | 0.55 |
| truthful information about diagnoses. | 3.49 (1.57) | 69.7 | 0.95 | 0.83 |  |
| treatment of vulnerable groups of patients. | 3.82 (1.37) | 76.3 | 0.95 | 0.86 |  |
| dealing with phobic patients. | 3.92 (1.39) | 78.4 | 0.95 | 0.84 |  |
| dealing with treatment errors. | 4.18 (1.22) | 83.6 | 0.96 | 0.76 |  |
| dealing with patients who have a lack of financial possibilities for the recommended therapy. | 3.89 (1.26) | 77.8 | 0.93 | 0.80 |  |
| dealing with conflicts between dental students and lecturer regarding the therapy decision. | 3.63 (1.45) | 72.6 | 0.96 | 0.80 |  |
| dealing with patients who can no longer make their own decisions. | 4.05 (1.21) | 81.0 | 0.93 | 0.86 |  |
| dealing with claims from patients after therapy without medical indication. | 3.82 (1.31) | 76.4 | 0.96 | 0.84 |  |
| dealing with parents who refuse to have their child treated. | 4.09 (1.27) | 81.9 | 0.93 | 0.76 |  |
| dealing with intercultural conflicts. | 3.38 (1.49) | 67.6 | 0.89 | 0.79 |  |
| dealing with wish-fulfilling treatment (i.e. aesthetic/cosmetic dentistry). | 3.60 (1.37) | 72.0 | 0.96 | 0.73 |  |
| dealing with stigmatisation. | 3.33 (1.50) | 66.6 | 0.91 | 0.82 |  |
| dealing with uncooperative behaviour. | 3.33 (1.60) | 66.6 | 0.97 | 0.73 |  |
| dealing with oversupply. | 3.60 (1.41) | 72.0 | 0.96 | 0.81 |  |
| dealing with the involvement of patient representatives. | 3.74 (1.39) | 74.7 | 0.96 | 0.83 |  |

SD standard deviation; KMO Kaiser-Meyer Olkin; ^#^ range from 0 – disagree entirely to 5 – agree completely; * Loading > 0.4 were reported

**Supplementary table 7: Dimension: Expectations and desires in terms of teaching medical ethics**

|  | **Mean (SD)** | **Item difficulty** | **KMO** | **Component loading** | | | | |
| --- | --- | --- | --- | --- | --- | --- | --- | --- |
|  |  |  |  | **Component 1** | **Component 2** | **Component 3** | **Component 4** | **Component 5** |
| **I would like the subject of medical ethics…** |  |  |  |  |  |  |  |  |
| to play an important role in dental education. | 3.21 (1.41) | 64.2 | 0.92 | 0.77 |  |  |  |  |
| to be an integral part of preclinical training. | 2.60 (1.72) | 51.9 | 0.87 | 0.64 |  |  |  |  |
| to be an integral part of clinical training. | 3.53 (1.54) | 70.6 | 0.94 | 0.66 |  |  |  |  |
| to deal with the lecturer-student situation. | 3.38 (1.48) | 67.5 | 0.91 |  | 0.76 |  |  |  |
| to deal with the student-patient situation. | 3.95 (1.32) | 78.9 | 0.94 | 0.66 |  |  |  |  |
| to deal with possible conflict situations between dental students and lecturer. | 3.35 (1.41) | 66.9 | 0.89 |  | 0.79 |  |  |  |
| to deal with possible conflict situations between students (e.g. the distributive justice of patients in courses). | 3.13 (1.61) | 62.6 | 0.89 |  | 0.82 |  |  |  |
| **I see the subject of medical ethics as…** |  |  |  |  |  |  |  |  |
| an important enrichment for my own knowledge growth. | 3.68 (1.40) | 73.7 | 0.92 | 0.74 |  |  |  |  |
| an important enrichment to my patient care. | 4.01 (1.23) | 80.3 | 0.91 | 0.74 |  |  |  |  |
| an important enrichment for collegial interaction. | 3.48 (1.48) | 69.5 | 0.90 |  | 0.65 |  |  |  |
| **The subject of medical ethics…** |  |  |  |  |  |  |  |  |
| should be taught by a professional medical ethicist. | 3.31 (1.28) | 66.2 | 0.85 |  | 0.45 |  |  |  |
| should be taught by a clinically experienced dentist. | 3.60 (1.16) | 72.0 | 0.62 |  |  |  |  | 0.87 |
| should be taught by a clinically experienced dentist who is trained in medical ethics. | 4.24 (0.99) | 84.8 | 0.86 |  |  |  |  | 0.66 |
| should be taught by a lecturer with dual qualifications (dentist and professional medical ethicist). | 3.82 (1.41) | 76.4 | 0.84 |  |  | 0.65 |  |  |
| should be taught in lectures. | 2.54 (1.60) | 50.8 | 0.70 |  |  |  | -0.71 |  |
| should be taught in small group courses. | 3.23 (1.72) | 64.6 | 0.84 |  |  |  | 0.74 |  |
| should be taught in POL groups (problem-oriented learning). | 2.97 (1.75) | 59.4 | 0.85 |  |  |  | 0.71 |  |
| should be taught through case studies. | 4.18 (1.13) | 83.6 | 0.94 |  |  | 0.52 |  |  |
| could be complemented by clinical ethics advice. | 3.32 (1.42) | 66.4 | 0.93 |  |  | 0.68 |  |  |
| could be supplemented by written teaching material. | 2.85 (1.68) | 57.0 | 0.88 |  |  | 0.62 |  |  |
| **I think it is important for…** |  |  |  |  |  |  |  |  |
| assistant dentists to be able to address ethical issues during the course. | 4.10 (1.03) | 82.0 | 0.85 |  |  | 0.57 |  |  |
| ethics lecturers to be available for a regular (e.g. weekly) visit to discuss questions that have arisen during the study course. | 3.28 (1.46) | 65.6 | 0.94 |  |  |  | 0.42 |  |

SD standard deviation; KMO Kaiser-Meyer Olkin; ^#^ range from 0– disagree entirely to 5 – agree completely; * Loading > 0.4 were reported
