# Supplementary material for: Identification of semester-specific teaching contents for dental ethics: development, testing and validation of a questionnaire
Source: BMC Med Educ. 2021 Feb 17;21:109. doi: 10.1186/s12909-021-02541-x (PMC7890951; doi:10.1186/s12909-021-02541-x)
Supplement: Supplementary file 2 — Additional file 2. [file 12909_2021_2541_MOESM2_ESM.docx]

**Identification of semester-specific teaching contents for dental ethics:**

**Development, testing and validation of a questionnaire**

**-**

**Supplementary information file: Questionnaire**

Katja Goetz^1^, Ann-Christine Gutermuth^2^, Hans-Jürgen Wenz^3^, Dominik Groß^4^, Katrin Hertrampf^2^

^1^Institute of Family Medicine

University Hospital Schleswig-Holstein, Campus Lübeck, Germany

^2^Clinic of Oral and Maxillofacial Surgery

University Hospital Schleswig-Holstein, Campus Kiel, Germany

^3^Clinic of Prosthodontics, Propaedeutics and Dental Materials

University Hospital Schleswig-Holstein, Campus Kiel, Germany

^4^Institute for History, Theory and Ethics of Medicine

University Hospital Aachen, RWTH Aachen University, Germany

**Dimension: Previous knowledge regarding ethical issues**

| **Please complete the appropriate category.** | Fully dis-agree |  |  |  |  | Fully agree |
| --- | --- | --- | --- | --- | --- | --- |
| So far, there have been no ethical conflicts within the family. | ❑ | ❑ | ❑ | ❑ | ❑ | ❑ |
| So far, there has been no situation during my studies in which ethics has played a role. | ❑ | ❑ | ❑ | ❑ | ❑ | ❑ |
| So far, there have been no ethical conflicts within my circle of friends. | ❑ | ❑ | ❑ | ❑ | ❑ | ❑ |
| Ethicists are unacquainted with regard to medical and clinical issues. | ❑ | ❑ | ❑ | ❑ | ❑ | ❑ |
| Ethicists cannot adequately evaluate treatment situations on patients due to a lack of personal treatment experience. | ❑ | ❑ | ❑ | ❑ | ❑ | ❑ |
| Ethicists set moral principles. | ❑ | ❑ | ❑ | ❑ | ❑ | ❑ |
| Morality is understood to mean social norms and values that guide the actions of an individual. | ❑ | ❑ | ❑ | ❑ | ❑ | ❑ |
| Ethics are social norms and values that guide the actions of society. | ❑ | ❑ | ❑ | ❑ | ❑ | ❑ |
| Ethics is a synonym for morality. | ❑ | ❑ | ❑ | ❑ | ❑ | ❑ |
| Professional ethics refers to the idea of values in terms of how society views a professional group. | ❑ | ❑ | ❑ | ❑ | ❑ | ❑ |
| Professional ethics describes individual moral aspects of a professional group. | ❑ | ❑ | ❑ | ❑ | ❑ | ❑ |
| Etiquette describes a set of rules of conduct that describes the expectations placed on the social behaviour of members of a professional group. | ❑ | ❑ | ❑ | ❑ | ❑ | ❑ |
| Etiquette is not about ethics. | ❑ | ❑ | ❑ | ❑ | ❑ | ❑ |

Definitions:

**Morality** refers to social norms or values that guide the actions of an individual, a social group, or a society.

**Ethics** is the scientific study of morality or the science of morality.

**Professional ethics** refers to individual values that are regarded as valid and identity-forming in a professional group (e.g. medical confidentiality). (This is about individual moral aspects).

(Professional) **etiquette** refers to a set of rules of conduct that describes the expectations for the social behaviour of members of a professional group (e.g. not making disparaging remarks about colleagues). (This is not about ethics, but about the professional group presenting a good image to the outside world).

The four principles of ethics are named below. Please read through the text and, in the three case descriptions that follow, name which principles can play a role in a decision.

**Principle ethics** (according to Beauchamp and Childress) is based on four principles that are derived from general morality ("common morality") and therefore enjoy a high level of acceptance - irrespective of personal ideological, religious, and/or cultural character.

- Respect for patient autonomy (recognition of patient self-determination)
- Non-maleficence (non-injury requirement)
- Beneficence (commandment of doing good)
- Justice (fair treatment, e.g. of patients and third parties, e.g. colleagues)

Description 1

A patient who has been very well informed by you absolutely wants anterior veneers. The veneers are to conceal minimal enamel spots.

| What kind of the four principles do you agree? | Disagreement | Agreement |
| --- | --- | --- |
| Respect for patient autonomy | ❑ | ❑ |
| Non-maleficence | ❑ | ❑ |
| Beneficience | ❑ | ❑ |
| Justice | ❑ | ❑ |

Description 2

During the group assignment for an oral exam in the prep course in anatomy, you happen to witness a conversation in which a fellow student tells another fellow student that he/she has objected to the instructor about the composition of his/her group. He/she believes that one person's very weak course performance up to that point could negatively affect the grades on the oral exam for all group members.

| What kind of the four principles do you agree? | Disagreement | Agreement |
| --- | --- | --- |
| Respect for patient autonomy | ❑ | ❑ |
| Non-maleficence | ❑ | ❑ |
| Beneficence | ❑ | ❑ |
| Justice | ❑ | ❑ |

Description 3

An elderly mentally fully oriented patient has an appointment at the outpatient clinic of oral and maxillofacial surgery to discuss the diagnosis of a tissue sample taken two weeks ago. She is accompanied by her husband. As the doctor approaches the treatment chair, he is intercepted by the husband and told not to tell his wife the truth in case of a possible malignant diagnosis.

| What kind of the four principles do you agree? | Disagreement | Agreement |
| --- | --- | --- |
| Respect for patient autonomy | ❑ | ❑ |
| Non-maleficence | ❑ | ❑ |
| Beneficence | ❑ | ❑ |
| Justice | ❑ | ❑ |

| **If you now reflect on this complex of questions in retrospect, to what extent do the following statements apply, from your point of view?** | Fully dis-agree |  |  |  |  | Fully agree |
| --- | --- | --- | --- | --- | --- | --- |
| Ethical conflicts have not played a role in my family so far. | ❑ | ❑ | ❑ | ❑ | ❑ | ❑ |
| There have been no situations during my studies in which ethics has played a role. | ❑ | ❑ | ❑ | ❑ | ❑ | ❑ |
| Within my circle of friends there have been no ethical conflicts so far. | ❑ | ❑ | ❑ | ❑ | ❑ | ❑ |

**Dimension: Dealing with ethical issues**

| **During my studies I was taught…** (Please complete the appropriate category.) | Fully dis-agree |  |  |  |  | Fully agree |
| --- | --- | --- | --- | --- | --- | --- |
| how to educate patients. | ❑ | ❑ | ❑ | ❑ | ❑ | ❑ |
| what dental confidentiality entails. | ❑ | ❑ | ❑ | ❑ | ❑ | ❑ |
| how to deal with vulnerable* patient groups during treatment. *Vulnerable patient groups include people due to their physical and/or mental constitution and/or due to their special social situation (disability, pregnancy, old age). | ❑ | ❑ | ❑ | ❑ | ❑ | ❑ |
| how to deal with phobic patients. | ❑ | ❑ | ❑ | ❑ | ❑ | ❑ |
| what a treatment error entails. | ❑ | ❑ | ❑ | ❑ | ❑ | ❑ |
| how to deal with a conflict between dental student and lecturer regarding the therapy decision. | ❑ | ❑ | ❑ | ❑ | ❑ | ❑ |
| how to deal with patients who can no longer make their own decisions. | ❑ | ❑ | ❑ | ❑ | ❑ | ❑ |
| how to deal with patients who have a lack of financial possibilities for the recommended therapy. | ❑ | ❑ | ❑ | ❑ | ❑ | ❑ |
| how to deal with claims from patients after therapy without medical indication. | ❑ | ❑ | ❑ | ❑ | ❑ | ❑ |
| how to deal with parents who refuse to have their child treated. | ❑ | ❑ | ❑ | ❑ | ❑ | ❑ |
| how to deal with intercultural* conflicts. *Conflicts between people from different cultural backgrounds | ❑ | ❑ | ❑ | ❑ | ❑ | ❑ |
| how to deal with stigmatisation*. *external characteristics (e.g. skin colour, visible disability) are assigned negative ratings | ❑ | ❑ | ❑ | ❑ | ❑ | ❑ |
| to inform about diagnoses truthfully. | ❑ | ❑ | ❑ | ❑ | ❑ | ❑ |
| how to deal with wish-fulfilling treatment (i.e. aesthetic/cosmetic dentistry). | ❑ | ❑ | ❑ | ❑ | ❑ | ❑ |
| that a dentist could be the head of a clinical trial. | ❑ | ❑ | ❑ | ❑ | ❑ | ❑ |
| how to deal with uncooperative behaviour. | ❑ | ❑ | ❑ | ❑ | ❑ | ❑ |
| how to deal with oversupply*. *(medical) oversupply is treatment that goes beyond what is therapeutically required and thus (potentially) harms the patient | ❑ | ❑ | ❑ | ❑ | ❑ | ❑ |
| how to deal with the involvement of patient representatives*. *Person (also groups) representing the interests of patients | ❑ | ❑ | ❑ | ❑ | ❑ | ❑ |

| **I think it is important that, during my studies, I am taught …**  (Please complete the appropriate category.) | Fully dis-agree |  |  |  |  | Fully agree |
| --- | --- | --- | --- | --- | --- | --- |
| about the education of patients. | ❑ | ❑ | ❑ | ❑ | ❑ | ❑ |
| about the observation of dental confidentiality. | ❑ | ❑ | ❑ | ❑ | ❑ | ❑ |
| about the truthful information of diagnoses. | ❑ | ❑ | ❑ | ❑ | ❑ | ❑ |
| about the treatment of vulnerable* groups of patients. *Vulnerable groups include people due to their physical and/or mental constitution and/or due to their special social situation (disability, pregnancy, old age). | ❑ | ❑ | ❑ | ❑ | ❑ | ❑ |
| about dealing with phobic patients. | ❑ | ❑ | ❑ | ❑ | ❑ | ❑ |
| about dealing with treatment errors. | ❑ | ❑ | ❑ | ❑ | ❑ | ❑ |
| about dealing with patients who have a lack of financial possibilities for the recommended therapy. | ❑ | ❑ | ❑ | ❑ | ❑ | ❑ |
| about dealing with patients who can no longer make their own decisions. | ❑ | ❑ | ❑ | ❑ | ❑ | ❑ |
| about dealing with conflict between dental students and lecturer regarding the therapy decision. | ❑ | ❑ | ❑ | ❑ | ❑ | ❑ |
| about dealing with intercultural* conflicts. *Conflicts between people from different cultural backgrounds | ❑ | ❑ | ❑ | ❑ | ❑ | ❑ |
| about dealing with claims from patients after therapy without medical indication. | ❑ | ❑ | ❑ | ❑ | ❑ | ❑ |
| about dealing with parents who refuse to have their child treated. | ❑ | ❑ | ❑ | ❑ | ❑ | ❑ |
| that a dentist can be the head of a clinical trial. | ❑ | ❑ | ❑ | ❑ | ❑ | ❑ |
| about dealing with wish-fulfilling treatment (i.e. aesthetic/cosmetic dentistry). | ❑ | ❑ | ❑ | ❑ | ❑ | ❑ |
| about dealing with stigmatisation*. *external characteristics (e.g. skin colour, visible disability) are assigned negative ratings | ❑ | ❑ | ❑ | ❑ | ❑ | ❑ |
| to deal with uncooperative behaviour. | ❑ | ❑ | ❑ | ❑ | ❑ | ❑ |
| about dealing with oversupply*. *(medical) oversupply is treatment that goes beyond what is therapeutically required and thus (potentially) harms the patient | ❑ | ❑ | ❑ | ❑ | ❑ | ❑ |
| about dealing with the involvement of patient representatives*. *Person (also groups) representing the interests of patients | ❑ | ❑ | ❑ | ❑ | ❑ | ❑ |

| **I have my own experience …** (Please complete the appropriate category.) | Fully dis-agree |  |  |  |  | Fully agree |
| --- | --- | --- | --- | --- | --- | --- |
| in educating patients. | ❑ | ❑ | ❑ | ❑ | ❑ | ❑ |
| in observing dental confidentiality. | ❑ | ❑ | ❑ | ❑ | ❑ | ❑ |
| with phobic patients. | ❑ | ❑ | ❑ | ❑ | ❑ | ❑ |
| with vulnerable* groups of patients. *Vulnerable groups include people due to their physical and/or mental constitution and/or due to their special social situation (disability, pregnancy, old age). | ❑ | ❑ | ❑ | ❑ | ❑ | ❑ |
| with treatment errors. | ❑ | ❑ | ❑ | ❑ | ❑ | ❑ |
| with patients who have a lack of financial possibilities for the recommended therapy. | ❑ | ❑ | ❑ | ❑ | ❑ | ❑ |
| in terms of conflict between dental students and lecturer regarding the therapy decision. | ❑ | ❑ | ❑ | ❑ | ❑ | ❑ |
| with patients who can no longer make their own decisions. | ❑ | ❑ | ❑ | ❑ | ❑ | ❑ |
| with claims from patients after therapy without medical indication. | ❑ | ❑ | ❑ | ❑ | ❑ | ❑ |
| with parents who refuse to have their child treated. | ❑ | ❑ | ❑ | ❑ | ❑ | ❑ |
| with intercultural* conflicts. *Conflicts between people from different cultural backgrounds | ❑ | ❑ | ❑ | ❑ | ❑ | ❑ |
| with a dentist who is the head of a clinical trial. | ❑ | ❑ | ❑ | ❑ | ❑ | ❑ |
| with stigmatisation*. *external characteristics (e.g. skin colour, visible disability) are assigned negative ratings | ❑ | ❑ | ❑ | ❑ | ❑ | ❑ |
| in dealing with truthfulness (e.g. information about diagnoses). | ❑ | ❑ | ❑ | ❑ | ❑ | ❑ |
| with wish-fulfilling treatment (i.e. aesthetic/cosmetic dentistry). | ❑ | ❑ | ❑ | ❑ | ❑ | ❑ |
| with uncooperative behaviour. | ❑ | ❑ | ❑ | ❑ | ❑ | ❑ |
| with oversupply*. *(medical) oversupply is treatment that goes beyond what is therapeutically required and thus (potentially) harms the patient | ❑ | ❑ | ❑ | ❑ | ❑ | ❑ |
| with the involvement of patient representatives*. *Person (also groups) representing the interests of patients | ❑ | ❑ | ❑ | ❑ | ❑ | ❑ |

| **I feel confident in…**  (Please complete the appropriate category.) | Fully dis-agree |  |  |  |  | Fully agree |
| --- | --- | --- | --- | --- | --- | --- |
| the education of patients. | ❑ | ❑ | ❑ | ❑ | ❑ | ❑ |
| the observation of dental confidentiality. | ❑ | ❑ | ❑ | ❑ | ❑ | ❑ |
| dealing with truthfulness (e.g. information about diagnoses). | ❑ | ❑ | ❑ | ❑ | ❑ | ❑ |
| the treatment of vulnerable* groups of patients. *Vulnerable groups include people due to their physical and/or mental constitution and/or due to their special social situation (disability, pregnancy, old age). | ❑ | ❑ | ❑ | ❑ | ❑ | ❑ |
| dealing with phobic patients. | ❑ | ❑ | ❑ | ❑ | ❑ | ❑ |
| dealing with treatment errors. | ❑ | ❑ | ❑ | ❑ | ❑ | ❑ |
| dealing with patients who have a lack of financial possibilities for the recommended therapy. | ❑ | ❑ | ❑ | ❑ | ❑ | ❑ |
| dealing with conflicts between dental students and lecturer regarding the therapy decision. | ❑ | ❑ | ❑ | ❑ | ❑ | ❑ |
| dealing with patients who can no longer make their own decisions. | ❑ | ❑ | ❑ | ❑ | ❑ | ❑ |
| dealing with claims from patients after therapy without medical indication. | ❑ | ❑ | ❑ | ❑ | ❑ | ❑ |
| dealing with parents who refuse to have their child treated. | ❑ | ❑ | ❑ | ❑ | ❑ | ❑ |
| dealing with intercultural* conflicts. *Conflicts between people from different cultural backgrounds | ❑ | ❑ | ❑ | ❑ | ❑ | ❑ |
| aesthetic/cosmetic dentistry. | ❑ | ❑ | ❑ | ❑ | ❑ | ❑ |
| dealing with stigmatisation*. *external characteristics (e.g. skin colour, visible disability) are assigned negative ratings | ❑ | ❑ | ❑ | ❑ | ❑ | ❑ |
| dealing with uncooperative behaviour. | ❑ | ❑ | ❑ | ❑ | ❑ | ❑ |
| dealing with oversupply*. *(medical) oversupply is treatment that goes beyond what is therapeutically required and thus (potentially) harms the patient | ❑ | ❑ | ❑ | ❑ | ❑ | ❑ |
| dealing with the involvement of patient representatives*. *Person (also groups) representing the interests of patients | ❑ | ❑ | ❑ | ❑ | ❑ | ❑ |

| **I would like support in terms of…**  (Please complete the appropriate category.) | Fully dis-agree |  |  |  |  | Fully agree |
| --- | --- | --- | --- | --- | --- | --- |
| the education of patients. | ❑ | ❑ | ❑ | ❑ | ❑ | ❑ |
| the implementation of dental confidentiality. | ❑ | ❑ | ❑ | ❑ | ❑ | ❑ |
| truthful information about diagnoses. | ❑ | ❑ | ❑ | ❑ | ❑ | ❑ |
| treatment of vulnerable* groups of patients. *Vulnerable groups include people due to their physical and/or mental constitution and/or due to their special social situation (disability, pregnancy, old age). | ❑ | ❑ | ❑ | ❑ | ❑ | ❑ |
| dealing with phobic patients. | ❑ | ❑ | ❑ | ❑ | ❑ | ❑ |
| dealing with treatment errors. | ❑ | ❑ | ❑ | ❑ | ❑ | ❑ |
| dealing with patients who have a lack of financial possibilities for the recommended therapy. | ❑ | ❑ | ❑ | ❑ | ❑ | ❑ |
| dealing with conflicts between dental students and lecturer regarding the therapy decision. | ❑ | ❑ | ❑ | ❑ | ❑ | ❑ |
| dealing with patients who can no longer make their own decisions. | ❑ | ❑ | ❑ | ❑ | ❑ | ❑ |
| dealing with claims from patients after therapy without medical indication. | ❑ | ❑ | ❑ | ❑ | ❑ | ❑ |
| dealing with parents who refuse to have their child treated. | ❑ | ❑ | ❑ | ❑ | ❑ | ❑ |
| dealing with intercultural* conflicts. *Conflicts between people from different cultural backgrounds | ❑ | ❑ | ❑ | ❑ | ❑ | ❑ |
| dealing with wish-fulfilling treatment (i.e. aesthetic/cosmetic dentistry). | ❑ | ❑ | ❑ | ❑ | ❑ | ❑ |
| dealing with stigmatisation*. *external characteristics (e.g. skin colour, visible disability) are assigned negative ratings | ❑ | ❑ | ❑ | ❑ | ❑ | ❑ |
| dealing with uncooperative behaviour. | ❑ | ❑ | ❑ | ❑ | ❑ | ❑ |
| dealing with oversupply*. *(medical) oversupply is treatment that goes beyond what is therapeutically required and thus (potentially) harms the patient | ❑ | ❑ | ❑ | ❑ | ❑ | ❑ |
| dealing with the involvement of patient representatives*. *Person (also groups) representing the interests of patients | ❑ | ❑ | ❑ | ❑ | ❑ | ❑ |

**Dimension: Expectations and desires in terms of teaching medical ethics**

| **I would like the subject of medical ethics…**  (Please complete the appropriate category.) | Fully dis-agree |  |  |  |  | Fully agree |
| --- | --- | --- | --- | --- | --- | --- |
| to play an important role in dental education. | ❑ | ❑ | ❑ | ❑ | ❑ | ❑ |
| to be an integral part of preclinical training. | ❑ | ❑ | ❑ | ❑ | ❑ | ❑ |
| to be an integral part of clinical training. | ❑ | ❑ | ❑ | ❑ | ❑ | ❑ |
| to deal with the lecturer-student situation. | ❑ | ❑ | ❑ | ❑ | ❑ | ❑ |
| to deal with the student-patient situation. | ❑ | ❑ | ❑ | ❑ | ❑ | ❑ |
| to deal with possible conflict situations between dental students and lecturer. | ❑ | ❑ | ❑ | ❑ | ❑ | ❑ |
| to deal with possible conflict situations between students (e.g. the distributive justice of patients in courses). | ❑ | ❑ | ❑ | ❑ | ❑ | ❑ |

| **I see the subject of medical ethics as…** (Please complete the appropriate category.) | Fully dis-agree |  |  |  |  | Fully agree |
| --- | --- | --- | --- | --- | --- | --- |
| an important enrichment for my own knowledge growth. | ❑ | ❑ | ❑ | ❑ | ❑ | ❑ |
| an important enrichment to my patient care. | ❑ | ❑ | ❑ | ❑ | ❑ | ❑ |
| an important enrichment for collegial interaction. | ❑ | ❑ | ❑ | ❑ | ❑ | ❑ |

| **The subject of medical ethics…**  (Please complete the appropriate category.) | Fully dis-agree |  |  |  |  | Fully agree |
| --- | --- | --- | --- | --- | --- | --- |
| should be taught by a professional medical ethicist. | ❑ | ❑ | ❑ | ❑ | ❑ | ❑ |
| should be taught by a clinically experienced dentist. | ❑ | ❑ | ❑ | ❑ | ❑ | ❑ |
| should be taught by a clinically experienced dentist who is trained in medical ethics. | ❑ | ❑ | ❑ | ❑ | ❑ | ❑ |
| should be taught by a lecturer with dual qualifications (dentist and professional medical ethicist). | ❑ | ❑ | ❑ | ❑ | ❑ | ❑ |
| should be taught in lectures. | ❑ | ❑ | ❑ | ❑ | ❑ | ❑ |
| should be taught in small group courses. | ❑ | ❑ | ❑ | ❑ | ❑ | ❑ |
| should be taught in POL groups (problem-oriented learning*). *Problem-oriented learning describes a teaching method in which students in small groups under moderation deal concretely and largely independently with case studies | ❑ | ❑ | ❑ | ❑ | ❑ | ❑ |
| should be taught through case studies. | ❑ | ❑ | ❑ | ❑ | ❑ | ❑ |
| could be complemented by clinical ethics advice*. *A counselling service aimed at patients, relatives, carers and doctors, advising them on possible ethical issues and conflicts when making decisions regarding medical treatments | ❑ | ❑ | ❑ | ❑ | ❑ | ❑ |
| could be supplemented by written teaching material. | ❑ | ❑ | ❑ | ❑ | ❑ | ❑ |

| **I think it is important for…**  (Please complete the appropriate category.) | Fully dis-agree |  |  |  |  | Fully agree |
| --- | --- | --- | --- | --- | --- | --- |
| assistant dentists to be able to address ethical issues during the course. | ❑ | ❑ | ❑ | ❑ | ❑ | ❑ |
| ethics lecturers to be available for a regular (e.g. weekly) visit to discuss questions that have arisen during the study course. | ❑ | ❑ | ❑ | ❑ | ❑ | ❑ |

Please add some sociodemographic data.

Gender ❑ female ❑ male

How old are you? _________ years

Semester of study? _________ semester

Do you work voluntary? ❑ yes ❑ no

Do you have a job in addition to your study? ❑ yes ❑ no

Do you have a filled in organ donor card? ❑ yes ❑ no
